# PTB/nPTB: master regulators of neuronal fate in mammals

**DOI:** 10.1007/s41048-018-0066-y

**Published:** 2018-08-28

**Authors:** Jing Hu, Hao Qian, Yuanchao Xue, Xiang-Dong Fu

**Affiliations:** 1 Department of Cellular and Molecular Medicine, University of California, La Jolla, San Diego, CA 92093-0651 USA; 2 Key Laboratory of RNA Biology, Institute of Biophysics, Chinese Academy of Sciences, Beijing, 100101 China

**Keywords:** Polypyrimidine tract-binding proteins, Auto- and cross-regulation of alternative splicing, MicroRNA, Neuronal fate determination

## Abstract

PTB was initially discovered as a polypyrimidine tract-binding protein (hence the name), which corresponds to a specific RNA-binding protein associated with heterogeneous ribonucleoprotein particle (hnRNP I). The PTB family consists of three members in mammalian genomes, with PTBP1 (PTB) expressed in most cell types, PTBP2 (also known as nPTB or brPTB) exclusively found in the nervous system, and PTBP3 (also known as ROD1) predominately detected in immune cells. During neural development, PTB is down-regulated, which induces nPTB, and the expression of both PTB and nPTB becomes diminished when neurons mature. This programed switch, which largely takes place at the splicing level, is critical for the development of the nervous system, with PTB playing a central role in neuronal induction and nPTB guarding neuronal maturation. Remarkably, sequential knockdown of PTB and nPTB has been found to be necessary and sufficient to convert non-neuronal cells to the neuronal lineage. These findings, coupled with exquisite understanding of the molecular circuits regulated by these RNA-binding proteins, establish a critical foundation for their future applications in regenerative medicine.

## Introduction

PTB was originally identified as an RNA-binding protein with strong sequence-specific binding preference for the pyrimidine-rich tract located between the branchpoint sequence and invariant AG dinucleotide, which together constitutes a functional 3′ splice site in pre-mRNA (Garcia-Blanco *et al*. 1989; Patton *et al*. 1991). PTB was soon recognized to correspond to hnRNP I in 2D gel that could be immunoprecipitated as part of the heterogeneous ribonucleoprotein particle (Wang and Pederson 1990). Because PTB showed association with the spliceosome, and under certain conditions, was able to complement the splicing reaction, it was initially thought to function as an essential splicing factor (Ghetti *et al*. 1992; Patton *et al*. 1991). This proves not to be the case (Lander *et al*. 2001), and for that matter, none of hnRNP proteins was later found to be essential for pre-mRNA splicing. However, PTB and nearly all hnRNP proteins have been shown to play roles in modulating splice site selection, thus functioning as splicing regulators in mammalian cells (Black 2003; Busch and Hertel 2012).

**Fig. 1:**
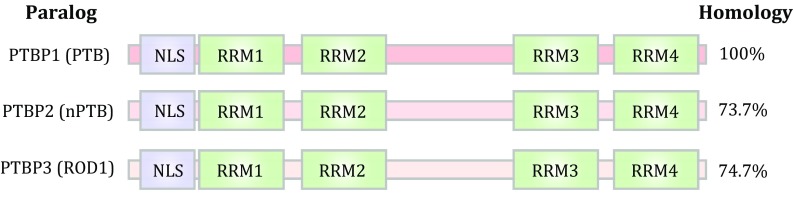
Domains and comparison of three PTB family members in the human genome. Each PTB paralog contains four RNA recognition motifs (RRMs) responsible for protein–RNA and protein–protein interactions, the latter of which is thought to mediate PTB dimerization or multimerization during splicing control

PTB has three paralogs in vertebrate genomes, sharing ~70% sequence homology among them at the protein level, each containing four RNA recognition motifs (RRMs) (Fig. 1). Biochemical and structural analysis showed that each RRM in PTB is able to independently bind RNA with similar preference for pyrimidine-rich motifs, suggesting the ability of PTB to create RNA looping (Oberstrass *et al*. 2005). Additionally, RRM3 and RRM4 are able to interact with one another, which may be important for its function as a dimer or multimer in splicing control (Spellman and Smith 2006). Importantly, each PTB family member is expressed in a highly tissue-specific manner: PTBP1 is largely ubiquitously expressed in most tissues and cell types except neurons, whereas its paralog PTBP2 is restricted to neurons, and thus is also known as nPTB or brain-specific brPTB (Ashiya and Grabowski 1997; Lillevali *et al*. 2001; Polydorides *et al*. 2000). The third paralog PTBP3 has been identified as regulator of differentiation 1 (ROD1) (Yamamoto *et al*. 1999). As little is known about the function and action mechanism of PTBP3/ROD1, we focus here on PTBP1 and PTBP2, and for simplicity, we refer to them as PTB and nPTB, respectively, throughout this review.

## Function of PTB in regulated splicing

The function of PTB in splicing control has been extensively studied on minigene models, which has been thoroughly reviewed earlier (Lander *et al*. 2001; Spellman and Smith 2006). Given its preference for pyrimidine-rich motifs, it was initially thought that PTB acted as a splicing repressor by directly competing with U2AF^65^, a *bona fide* essential splicing factor that binds the polypyrimidine tract to initiate spliceosome assembly (Lin and Patton 1995; Singh *et al*. 1995). However, additional analysis revealed that PTB often binds elsewhere in pre-mRNA (Lander *et al*. 2001). Detailed mechanistic studies on the alternative *c*-*src* exon N1, which is normally excluded in non-neuronal cells, but included in neurons, demonstrated that PTB binds flanking intronic regions of N1 to interfere with the communication of this alternative exon with the downstream functional 3′ splice site (Amir-Ahmady *et al*. 2005; Chou *et al*. 2000; Sharma *et al*. 2005). On the *Fas* exon 6 minigene, however, PTB was found to bind within the alternative exon to prevent the recognition of functional splice sites on both sides of the alternative exon by the splicing machinery, which may result from PTB multimerization to create a silencing zone across the exon (Izquierdo *et al*. 2005).

**Fig. 2:**
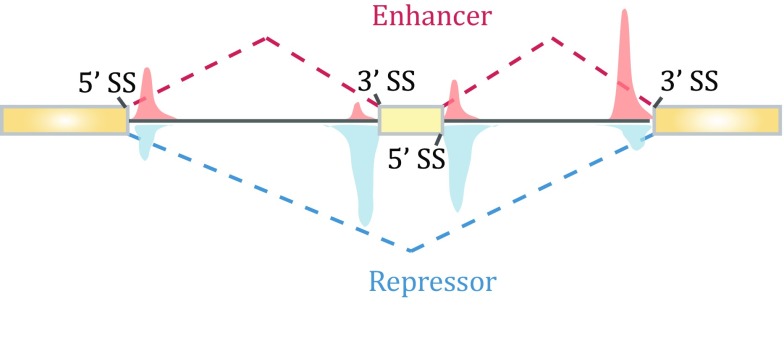
Position-dependent effects of PTB on regulated splicing. Compiling PTB-binding events on PTB-dependent exon inclusion events (*red*) versus PTB-dependent exon skipping events (*blue*) suggests that PTB action on flanking constitutive splice sites enhances the inclusion of the alternative exon in the middle, while PTB binding on or around the alternative exon causes skipping of the exon

In the post-genome era, the function of PTB in splicing control has studied at the genome scale. Its binding profile was first determined in HeLa cells by crosslinking immunoprecipitation sequencing (CLIP) (Xue *et al*. 2009). Interestingly, although PTB has been widely accepted as a splicing repressor, depletion of PTB in mammalian cells induced not only exon inclusion, as expected, but also exon skipping (Llorian *et al*. 2010; Xue *et al*. 2009). Integrated analysis of PTB binding and PTB-dependent splicing then revealed the position-dependent effect of PTB, which now applies to a large number of splicing regulators in mammalian cells (Corrionero and Valcarcel 2009; Fu and Ares 2014). In the case of PTB, such “functional splicing map” showed that PTB represses exon selection (thus its depletion causes exon inclusion) when it binds exonic and/or flanking intronic sequences around the alternative exon, whereas it promotes inclusion of many alternative exons (thus its depletion induces exon skipping) when it binds close to upstream constitutive 5′ splice site and/or downstream constitutive 3′ splice site, thereby strengthening the recognition of the alternative exon in-between (Fig. 2).

Relative to PTB, much less is known about the role of nPTB in regulated splicing, and whether PTBP3/ROD1 plays a role in regulated splicing has remained an open question. In general, it is thought that nPTB functions similarly as PTB in splicing control, but as a weaker splicing repressor, in the nervous system because nPTB seems to be less effective than PTB in rescuing splicing defects of multiple splicing substrates examined in PTB and nPTB double-depleted cells (Boutz *et al*. 2007b; Makeyev *et al*. 2007; Spellman *et al*. 2007). While the functional similarity between PTB and nPTB in splicing control is firm, as demonstrated by using PTB to replace nPTB in mature neurons in mice, these two PTB paralogs clearly have distinct functions in the development of the nervous system because PTB cannot fully rescue the phenotype caused by depletion of nPTB in developing neurons (Vuong *et al*. 2016b). Such functional distinction requires further investigation, as PTB and nPTB may enlist distinct co-factors in splicing regulation or have entirely distinct functions beyond splicing control in different tissues or cell types.

## Auto-regulation and cross-regulation of PTB family members

Depleting PTB or nPTB in cellular and animal models causes widespread changes in alternative splicing of a large number of genes (Coutinho-Mansfield *et al*. 2007; Vuong *et al*. 2016a). Because nPTB is exclusively expressed in the brain, its depletion has been found to affect a large array of neuronal-specific genes (Gueroussov *et al*. 2015), including PSD-95 in mature neurons critical for excitatory synapse formation (Zheng *et al*. 2012), and thus, such splicing defects are widely thought to contribute to specific neuronal phenotypes. Given so many splicing events are affected, however, it has been a major challenge in linking specific altered splicing events to defined phenotypic differences in most cases. Interestingly, sequential expression of PTB and nPTP during neuronal differentiation and characterization of their own regulated splicing programs have led to an elegant demonstration for auto- and cross-regulation among PTB family members that are of direct functional relevance to the development of the nervous system (Boutz *et al*. 2007b; Makeyev *et al*. 2007; Spellman *et al*. 2007).

**Fig. 3:**
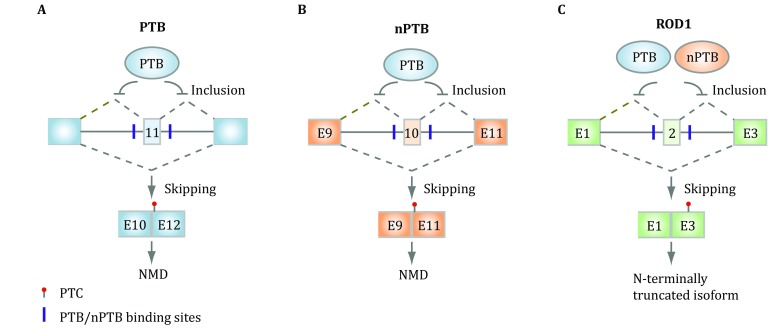
Auto- and cross-regulation of PTB family members at the splicing level. **A** PTB binds the flanking intronic sequences of the alternative exon 11 to auto-regulate its own expression, as exclusion of this exon will trigger NMD. **B** PTB also represses the inclusion of the alternative exon 10, which is required to generate full-length mRNA for nPTB in non-neuronal cells. PTB down-regulation would thus induce this exon to produce functional nPTB during neuronal development. **C** PTB and nPTB appear to function in a redundant fashion to repress the inclusion of the alternative exon 2 in PTBP3/ROD1, but the functional significance of this regulated splicing event remains to be determined

It turns out that all three PTB family members themselves undergo alternative splicing in mammalian cells. In particular, PTB contains an alternative exon 11. PTB binds flanking intronic regions of this highly conserved alternative exon for its auto-regulation in non-neuronal cells (Fig. 3A). This is because PTB represses the inclusion of exon 11 whose exclusion will alter the reading frame to create a premature termination codon (PTC) in exon 12, which will trigger nonsense-mediated mRNA decay (NMD) (Wollerton *et al*. 2004). It has been estimated that NMD consumes ~20% of PTB mRNA due to the suppression of its own exon 11. This ensures homeostatic expression of PTB in non-neuronal cells because increased PTB expression will reduce the inclusion of exon 11 to trigger NMD to reduce PTB expression, whereas decreased PTB expression will enhance the inclusion of exon 11 to produce more full-length mRNA to increase PTB expression. In developing neurons, however, PTB is progressively down-regulated due to the induction of the neuronal-specific microRNA miR-124 (Boutz *et al*. 2007b; Makeyev *et al*. 2007).

Similar to PTB, nPTB also carries an alternative exon 10, which is repressed by PTB in non-neuronal cells. As this exon is required to express a full-length functional nPTB, skipping of this alternative exon would prevent the expression of nPTB, as the resulting mRNA will be sensitive to NMD (Fig. 3B). This affords the induction of nPTB when PTB expression is diminished in developing neurons, leading to progressive elevation of nPTB expression while PTB expression is gradually reduced. This cross-regulation demonstrates an elegant post-transcriptional strategy to switch the expression from PTB to nPTB during development of the nervous system, which proves to be functionally required (Coutinho-Mansfield *et al*. 2007).

Last, but not least, PTBP3/ROD1 also carries an alternative exon 2, which is repressed by both PTB and nPTB, and the exclusion has been shown to introduce a stop codon in exon 3, thus may generate a potentially very short peptide (Fig. 3C) (Spellman *et al*. 2007). As PTBP3/ROD1 is mainly expressed in the immune system, it will be interesting to determine whether the development of the immune system also enlists a related strategy, as hinted by the name of ROD1. Together, such post-transcriptional strategy is responsible, in part, for tissue-specific expression of different PTB paralogs encoded in mammalian genomes.

## Roles of PTB beyond splicing control

Besides its established functions in splicing control, PTB has been implicated in other processes of regulated gene expression, including polyadenylation (Castelo-Branco *et al*. 2004; Moreira *et al*. 1998), translation (Mitchell *et al*. 2005), and mRNA stability (Hamilton *et al*. 2003; Pautz *et al*. 2006). Relative to splicing control, however, our mechanistic understanding of such regulations has remained primitive. An intriguing possibility is that some of these splicing-independent functions of PTB may result from the role of PTB in antagonizing microRNA function, thereby modulating mRNA stability, as demonstrated during PTB-depletion-induced neurogenesis (Xue *et al*. 2013).

**Fig. 4:**
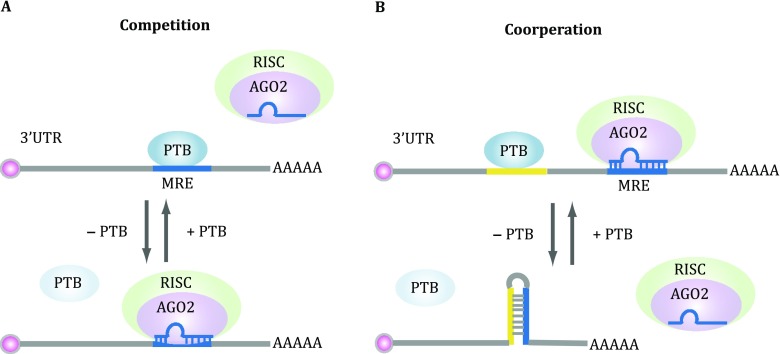
Modulation of microRNA targeting by PTB. **A** PTB binding directly competes with microRNA targeting on the microRNA responsive element (MRE). PTB depletion will thus promote microRNA targeting, thus enhancing microRNA-mediated post-transcriptional repression of gene expression. **B** PTB binding may also disrupt RNA secondary structure, which shields some MREs. PTB binding would expose such MREs for microRNA targeting, thereby enhancing post-transcriptional silencing of those genes

It has been well established that PTB is targeted by miR-124 during neuronal differentiation (Makeyev *et al*. 2007), and nPTB by miR-133 during muscle differentiation (Boutz *et al*. 2007a). Interestingly, based on the PTB CLIP data generated on HeLa cells (Xue *et al*. 2013), it is clear that PTB also finds pyrimidine-rich sequences in 3′UTR of various genes, some of which are coincident with specific microRNA responsive elements (Fig. 4A). Indeed, by using reporter-based assays and through mapping Ago2 occupancy before and after PTB depletion in the genome, it became clear that PTB is able to directly compete with targeting of specific microRNAs on 3′UTR (Xue *et al*. 2013). Therefore, in the absence of PTB, those microRNAs become more efficient in inducing mRNA degradation and/or translational repression, which may also account for the observed effect of PTB in promoting insulin secretory granule biogenesis in β-cells (Knoch *et al*. 2004). Further interesting is the observation that PTB depletion not only destabilizes certain target mRNAs, but also exerts the opposite effect on others (Xue *et al*. 2013). This turns out to be due to its role in modulating RNA secondary structure (Fig. 4B). In certain cases, PTB binding disrupts such secondary structure to expose specific microRNA target sequences for their recognition by the microRNA machinery, and when PTB is depleted, such secondary structure is restored to prevent microRNA targeting, leading to mRNA stabilization. Together, these data suggest that PTB is both target and modulator of specific microRNAs in the cell, thereby building up additional layers in its regulated gene network during neuronal differentiation. It would be intriguing to investigate whether nPTB and ROD1 have similar microRNA modulation functions in future studies.

## PTB/nPTB switch during development of the nervous system

PTB is expressed in most cell types. Consistent with the ubiquitous expression of PTB and its broad function in regulated gene expression, genetic ablation of PTB resulted in embryonic lethality (Shibasaki *et al*. 2013). Conditional knockout of PTB in the brain caused precocious neurogenesis of radial glial cells, a precursor cell type that gives rise to multiple neuronal cell lineages in the nervous system (Gotz and Barde 2005). This phenotype is actually related to the neuronal progenitor depletion phenotype caused by genetic inactivation of REST, a well-known master negative regulator of neurogenesis (Gao *et al*. 2011; Singh *et al*. 2008), thus in line with a key role of regulated PTB expression in development of the nervous system. Functional and mechanistic studies of PTB have been further pursued in embryonic stem cells and neuronal progenitor cells upon induction to the neuronal lineage, during which PTB expression is diminished and nPTB induced due to their cross-regulation (Boutz *et al*. 2007b; Makeyev *et al*. 2007). PTB is down-regulated by the induction of neuronal-specific microRNA miR-124, and such down-regulation has been found to be essential for neuronal induction, as demonstrated by the ability of a constitutively expressed exogenous PTB to block the process (Makeyev *et al*. 2007).

Compared to the function of PTB in regulating neuronal induction, nPTB appears critical for neuronal maturation, as genetic ablation of this gene in the brain only compromised postnatal survival of knockout mice (Li *et al*. 2014; Licatalosi *et al*. 2012). A large number of adult neuron-specific alternative splicing events were affected in the developing brain, one of which characterized in detail corresponds to PSD-95, a key scaffold protein of excitatory synapses in mature neurons (Zheng *et al*. 2012). Thus, the developmental switch from PTB to nPTB likely drives a programed switch of splicing control in development of the nervous system (Vuong *et al*. 2016a). More recently, forced expression of PTB in nPTB null mice showed that PTB was able to rescue the phenotype of nPTB null mutation in forebrain, but not pan-neuronal knockout of nPTB in the brain (Vuong *et al*. 2016b), implying that PTB can largely replace the function of nPTB once neurons are fully matured, but not during the process of neuronal maturation. Although it has been postulated based on analysis of a set of PTB target genes that nPTB may act as a weaker splicing repressor than PTB (Makeyev *et al*. 2007), the non-redundant function of nPTB in developing neurons raises the possibility that PTB and nPTB may have distinct targets, perhaps via different co-factors, as postulated (Vuong *et al*. 2016b). It is also possible that nPTB may have functions beyond splicing control to drive neuronal maturation.

## Subtracting PTB and nPTB to trans-differentiate non-neuronal cells to neurons

**Fig. 5:**
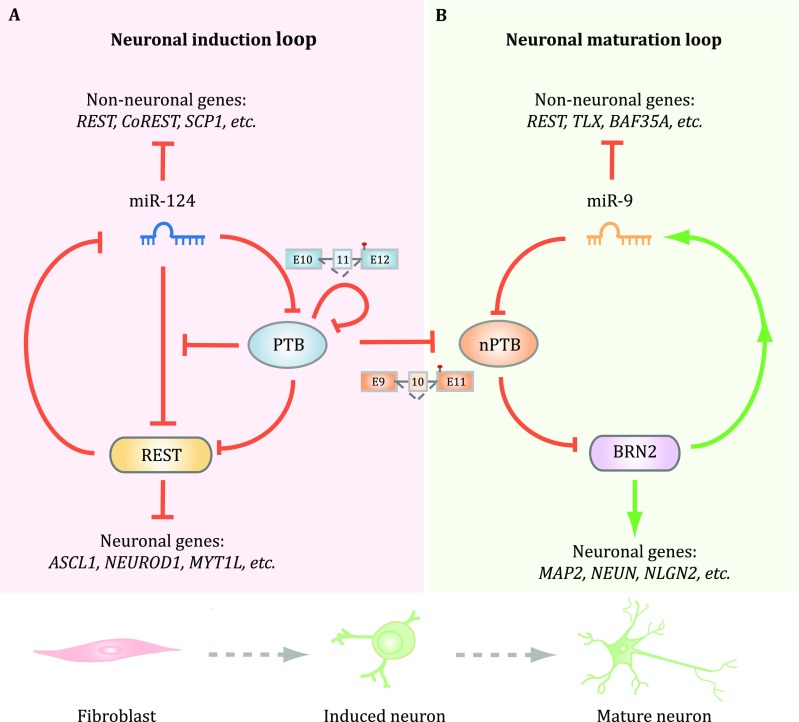
Regulation of two consecutive regulatory loops for neuronal induction and maturation by PTB and nPTB. **A** PTB functions as a breaker to miR-124 targeting of REST. Once PTB down-regulation is initiated, miR-124 becomes more efficient in targeting REST, and reduced REST further de-represses miR-124. This loop is self-enforced by two mechanisms because PTB itself is a target for miR-124 and reduced PTB also results in the inclusion of exon 11, together causing progressive PTB down-regulation during neuronal induction. **B** Reduced PTB expression de-represses nPTB due to the inclusion of the alternative exon 10. During neuronal maturation, induced miR-9 targets nPTB, which somehow leads to the induction of mature neuronal-specific transcription factor Brn2, and activated Brn2 further induces miR-9. Thus, PTB and nPTB function as two separate gatekeepers for neuronal induction and maturation. The two regulated loops are efficiently connected in mouse cells, likely because of high-level induction of miR-124, which is also capable of targeting nPTB, but the two loops have to be separately activated in human cells to generate functional neurons

It has been demonstrated that certain key lineage-specific transcription factors (TFs) are able to drive the development of the nervous system, as various combinations of those TFs are able to not only induce neuronal progenitors to differentiate into functional neurons, but also convert non-neuronal cells to the neuronal lineage (Vierbuchen and Wernig 2012). Remarkably, diminishing PTB expression alone was found to be sufficient to initiate the entire neuronal differentiation program in diverse cell types of mouse origin (Xue *et al*. 2013). Although diminished PTB expression is responsible for inducing a key neuronal-specific transcription factor Pbx1 through a splicing depression mechanism (Linares *et al*. 2015), the vast majority of neuronal lineage-specific TFs appears to be induced through inactivating the REST complex (Fig. 5A), thus qualifying PTB as a master regulator of the nervous system.

Mechanistically, non-neuronal cells are kept from becoming neurons by a negative feedback loop in which REST prevents the expression of a large number of neuronal-specific TFs as well as miR-124 (Xue *et al*. 2013). Interestingly, while miR-124 is able to target REST in such loop, such targeting is potently suppressed by PTB in non-neuronal cells via direct competition of miR-124 targeting on multiple REST components, including a key REST subunit SCP1, a Pol II phosphatase (Xue *et al*. 2013; Yeo *et al*. 2005). Thus, the presence of PTB prevents the effect of precociously induced miR-124. During neuronal induction, increased miR-124, which likely results from signal-induced transcription in the brain, will target PTB to reduce its expression. Therefore, when PTB inactivation is experimentally induced by RNAi, such negative REST/miR-124 loop is converted to a positive one to allow miR-124 to be more efficient in targeting REST and reduced REST further de-represses miR-124. In mouse cells, once this loop is activated, cells are progressively converted to functional neurons (Xue *et al*. 2013).

When the same approach was applied to human adult fibroblasts, however, PTB inactivation by RNAi was found to be sufficient to propel neuronal induction, but insufficient to drive the process all the way to functional neurons (Xue *et al*. 2016). This turns out to be due to persistent expression of nPTB in the absence of PTB in human cells, contrary to mouse cells in which nPTB is first induced and then diminished. Further analysis revealed another regulatory loop in which nPTB suppresses (directly or indirectly) the mature neuron-specific transcription factor Brn2, which activates miR-9, and this mature neuron-specific microRNA in turn targets nPTB (Fig. 5B). Once this second loop is activated by sequential knockdown of PTB and nPTB or PTB knockdown in combination of Brn2 overexpression, all trans-differentiated neurons from human adult fibroblasts become fully matured in the presence of co-cultured glial cells, showing all expected neuronal activities, including voltage-dependent Na^+^ currents, repetitive action potentials, and both excitatory and inhibitory synaptic currents (Xue *et al*. 2016).

Collectively, the experiments described above not only reveal the mechanism for nPTB down-regulation, but also suggest that the PTB-regulated REST/miR-124 loop is automatically connected to the second nPTB-regulated Brn2/miR-9 loop in mouse cells, but such two loops have to be separately activated in human adult fibroblasts. Such tight connection between the two loops in mouse, but not human cells might result from high-level activity of miR-124 in mouse, but not human cells because both miR-124 and miR-9 have been found to be capable of targeting nPTB (Xue *et al*. 2016). The beauty of these regulatory networks by PTB and nPTB lies in the utilization of multiple regulatory mechanisms at both transcriptional and post-transcriptional levels, rather than simple feedback and/or feed forward controls among TFs. The PTB/nPTP-mediated neuronal reprogramming also emphasizes the “subtraction” approach through inactivating negative master regulators compared to the general “addition” approach widely used in the regenerative medicine field via overexpressing positive master regulators.

## Advantage of multitasking PTB in cell fate determination

Given that PTB is part of the regulatory loop to induce neuronal differentiation, such loop could be, at least in theory, induced by modulating any component in the loop, *e.g.,* inactivating REST or overexpressing miR-124, to achieve the same end purpose. As a matter of fact, inactivating REST or its key component SCP1 has been shown to induce neuronal-specific gene expression program (Yeo *et al*. 2005). However, genetic ablation of REST has demonstrated that the complete loss of REST causes the depletion of neuronal progenitors and impairs viability of differentiated neurons (Gao *et al*. 2011). Therefore, REST has to be maintained at an adequate lower level in differentiated neurons. As a result, the efficiency of siRNA against REST has to be titrated in trans-differentiation, as too low would be insufficient to achieve neuronal conversation, whereas too high would be detrimental to the viability of converted neurons. It is currently unclear how to experimentally reduce REST to an optimal level to induce neurogenesis. Thus, the strategy to down-regulate REST yet keep down-regulated REST at the required low level is best achieved through the activation of the internal regulatory mechanism to maintain its hemostatic expression in differentiated neurons, such as the strategy of PTB knockdown.

Similarly, overexpressing miR-124 would lead to PTB down-regulation, thus activating the REST-miR-124 loop. However, it has been shown that miR-124 overexpression alone is insufficient to convert fibroblast to neurons (Yoo *et al*. 2011). Even in neural stem cells (*e.g.,* N2a cells), overexpressed miR-124 is able to enhance neuronal differentiation induced by RA, but itself is not sufficient to drive the neuronal differentiation program (Makeyev *et al*. 2007). This might be due to at least two technical reasons. First, in the published work on PTB knockdown-induced neurogenesis, PTB inactivation by siRNA is sufficient to trans-differentiate fibroblast into neurons, but the efficiency remains relative low, especially in human cells, which needs enhancement by a few small molecule inhibitors (Xue *et al*. 2016). Additionally, as microRNA is not as efficient as siRNA in gene silencing in general, it is possible that transfected miR-124 alone is inefficient in down-regulating PTB to initiate the cell fate switch to the neuronal lineage. As optimized pools of small molecule inhibitors have been shown to be sufficient in inducing cell fate switch to different lineages (Ebrahimi 2016; Gao *et al*. 2017), including from fibroblasts into neurons (Hu *et al*. 2015; Li *et al*. 2015), it is entirely possible that transfected miR-124 in combination with a cocktail of carefully titrated small molecular inhibitors might be able to drive neurogenesis.

For future therapeutic applications, especially in the brain, PTB/nPTB down-regulation appears to have multiple advantages over other strategies to induce neurogenesis. Because siRNA- or miRNA-based strategies suffer from challenging delivery problems, which has proven to be quite difficult in penetrating the brain blood barrier (BBB) (Mathupala 2009), the PTB inactivation-based strategy may bypass such technical problem by using ASO, which has been shown to effectively penetrate the BBB (Schoch and Miller 2017). Besides local delivery to minimize side effects, one can also envision additional ways to enhance the specificity by conjugating such anti-PTB ASO with a specific polypeptide to target desired cell types (Dowdy 2017). Thus, the PTB-based strategy may also be superior to small molecule-based approach for future applications *in vivo*, as the cell-type specificity of latter would be more difficult to manage. Because inactivation is clearly easier to achieve than overexpression, the “subtraction” approach would be more feasible to implement than the “addition” approach using TFs in inducing trans-differentiation *in vivo*, despite the success in using various overexpressed TFs to directly convert non-neuronal cells, particularly astrocytes, to functional neurons in mouse brain (Grande *et al*. 2013; Guo *et al*. 2014; Liu *et al*. 2015; Niu *et al*. 2013; Su *et al*. 2014; Torper *et al*. 2013), which may offer considerable advantage over cell-based approaches in future therapeutic applications (Goldman 2016). As the development and differentiation of a neuron likely proceeds through its due course to orchestrate a series of switches during cellular reprogramming, PTB inactivation merely triggers the course to let the internal transcriptional and post-transcriptional programs to develop and progress. By contrast, forced expression of a TF or a combination of TFs may either be insufficient if not sufficiently overexpressed or detrimental to the internal program if excessively forced.

## A parallel of PTB/nPTB-regulated circuitry in the muscle system?

PTB and nPTB have now been demonstrated to function as master regulators in the nervous system. Do these regulators also play roles in cell fate determination in other tissues? In fact, it has been found earlier that both PTB and nPTB are expressed in muscles and testis (Boutz *et al*. 2007a). Parallel to the nervous system, PTB is highly expressed in myoblasts and its expression declines during muscle differentiation during which nPTB is induced, and interestingly, nPTB has also been found to subject to down-regulation by induced muscle-specific miR-133, again parallel to its down-regulation by miR-9 in the nervous system (Xue *et al*. 2016). A more recent single-cell transcriptome analysis also revealed a critical role of PTB as a key barrier to conversion from cardiac fibroblasts to cardiomyocytes (Liu *et al*. 2017), although it is still under intensive debate in the cardiac field on whether cardiomyocytes are able to rise from cells of true non-cardiac linages, which has been argued against by using a rigorous double lineage tracing strategy (He *et al*. 2017). It is also of interest to note that such a deterministic role of miR-133 in muscle differentiation has remained an open question, as an early study showed that this microRNA was able to enhance muscle differentiation in C2C12 cells (Chen *et al*. 2006), but such effect was not observed in a separate study (Boutz *et al*. 2007a), which suggests a fine-tuning role, rather than a deterministic function, of miR-133 in enhancing the function of differentiated muscles via a series of regulated alternative splicing events. This is reminiscent of a parallel enhancing, rather than deterministic function of miR-124 in neuronal stem cells.

REST is well known to dampen the expression of a large number of neuronal-specific genes in non-neuronal cells. It is interesting to note that NRF2 might fulfill such role as a master negative regulator in the muscle system, as it has been shown to repress some key muscle-specific genes, such as MyoD (Whitman *et al*. 2013). As NRF2 has been well established to subject to a negative control by a dedicated ubiquitination system (Kansanen *et al*. 2013), it is curious to investigate whether and how NRF2 down-regulation might be connected to diminished PTB expression during muscle differentiation. Similarly, we know little about nPTB with respect to its potential contribution to muscle differentiation. Considered together, it appears that there seems to be a parallel PTB/nPTB-regulated circuitry in the muscle system, which clearly requires further investigation, and when properly developed, such circuitry might be developed as tools to learn muscle fate determination and to engineer trans-differentiation from non-muscle cells to muscle or to replenish lost cardiomyocytes in failing heart.

Last, but not the least, given the success in elucidating PTB- and nPTB-regulated gene networks in cell fate determination in the nervous system, the question is whether other RNA-binding proteins or families of them play similar roles in other cellular reprogramming processes. In this regard, the toolbox of ~1500 RNA-binding proteins expressed in mammalian cells (Gerstberger *et al*. 2014) remains to be explored.

## References

[CR1] Amir-Ahmady B, Boutz PL, Markovtsov V, Phillips ML, Black DL (2005). Exon repression by polypyrimidine tract binding protein. RNA.

[CR2] Ashiya M, Grabowski PJ (1997). A neuron-specific splicing switch mediated by an array of pre-mRNA repressor sites: evidence of a regulatory role for the polypyrimidine tract binding protein and a brain-specific PTB counterpart. RNA.

[CR3] Black DL (2003). Mechanisms of alternative pre-messenger RNA splicing. Annu Rev Biochem.

[CR4] Boutz PL, Chawla G, Stoilov P, Black DL (2007). MicroRNAs regulate the expression of the alternative splicing factor nPTB during muscle development. Genes Dev.

[CR5] Boutz PL, Stoilov P, Li Q, Lin CH, Chawla G, Ostrow K, Shiue L, Ares M, Black DL (2007). A post-transcriptional regulatory switch in polypyrimidine tract-binding proteins reprograms alternative splicing in developing neurons. Genes Dev.

[CR6] Busch A, Hertel KJ (2012). Evolution of SR protein and hnRNP splicing regulatory factors. Wiley Interdiscip Rev RNA.

[CR7] Castelo-Branco P, Furger A, Wollerton M, Smith C, Moreira A, Proudfoot N (2004). Polypyrimidine tract binding protein modulates efficiency of polyadenylation. Mol Cell Biol.

[CR8] Chen JF, Mandel EM, Thomson JM, Wu Q, Callis TE, Hammond SM, Conlon FL, Wang DZ (2006). The role of microRNA-1 and microRNA-133 in skeletal muscle proliferation and differentiation. Nat Genet.

[CR9] Chou MY, Underwood JG, Nikolic J, Luu MH, Black DL (2000). Multisite RNA binding and release of polypyrimidine tract binding protein during the regulation of c-src neural-specific splicing. Mol Cell.

[CR10] Corrionero A, Valcarcel J (2009). RNA processing: redrawing the map of charted territory. Mol Cell.

[CR11] Coutinho-Mansfield GC, Xue Y, Zhang Y, Fu XD (2007). PTB/nPTB switch: a post-transcriptional mechanism for programming neuronal differentiation. Genes Dev.

[CR12] Dowdy SF (2017). Overcoming cellular barriers for RNA therapeutics. Nat Biotechnol.

[CR13] Ebrahimi B (2016). Chemicals as the sole transformers of cell fate. Int J Stem Cells.

[CR14] Fu XD, Ares M (2014). Context-dependent control of alternative splicing by RNA-binding proteins. Nat Rev Genet.

[CR15] Gao Z, Ure K, Ding P, Nashaat M, Yuan L, Ma J, Hammer RE, Hsieh J (2011). The master negative regulator REST/NRSF controls adult neurogenesis by restraining the neurogenic program in quiescent stem cells. J Neurosci.

[CR16] Gao L, Guan W, Wang M, Wang H, Yu J, Liu Q, Qiu B, Yu Y, Ping Y, Bian X, Shen L, Pei G (2017). Direct generation of human neuronal cells from adult astrocytes by small molecules. Stem Cell Rep.

[CR17] Garcia-Blanco MA, Jamison SF, Sharp PA (1989). Identification and purification of a 62,000-dalton protein that binds specifically to the polypyrimidine tract of introns. Genes Dev.

[CR18] Gerstberger S, Hafner M, Tuschl T (2014). A census of human RNA-binding proteins. Nat Rev Genet.

[CR19] Ghetti A, Pinol-Roma S, Michael WM, Morandi C, Dreyfuss G (1992). hnRNP I, the polypyrimidine tract-binding protein: distinct nuclear localization and association with hnRNAs. Nucleic Acids Res.

[CR20] Goldman SA (2016). Stem and progenitor cell-based therapy of the central nervous system: hopes, hype, and wishful thinking. Cell Stem Cell.

[CR21] Gotz M, Barde YA (2005). Radial glial cells defined and major intermediates between embryonic stem cells and CNS neurons. Neuron.

[CR22] Grande A, Sumiyoshi K, Lopez-Juarez A, Howard J, Sakthivel B, Aronow B, Campbell K, Nakafuku M (2013). Environmental impact on direct neuronal reprogramming *in vivo* in the adult brain. Nat Commun.

[CR23] Gueroussov S, Gonatopoulos-Pournatzis T, Irimia M, Raj B, Lin ZY, Gingras AC, Blencowe BJ (2015). An alternative splicing event amplifies evolutionary differences between vertebrates. Science.

[CR24] Guo Z, Zhang L, Wu Z, Chen Y, Wang F, Chen G (2014). *In vivo* direct reprogramming of reactive glial cells into functional neurons after brain injury and in an Alzheimer’s disease model. Cell Stem Cell.

[CR25] Hamilton BJ, Genin A, Cron RQ, Rigby WF (2003). Delineation of a novel pathway that regulates CD154 (CD40 ligand) expression. Mol Cell Biol.

[CR26] He L, Li Y, Li Y, Pu W, Huang X, Tian X, Wang Y, Zhang H, Liu Q, Zhang L, Zhao H, Tang J, Ji H, Cai D, Han Z, Han Z, Nie Y, Hu S, Wang QD, Sun R, Fei J, Wang F, Chen T, Yan Y, Huang H, Pu WT, Zhou B (2017). Enhancing the precision of genetic lineage tracing using dual recombinases. Nat Med.

[CR27] Hu W, Qiu B, Guan W, Wang Q, Wang M, Li W, Gao L, Shen L, Huang Y, Xie G, Zhao H, Jin Y, Tang B, Yu Y, Zhao J, Pei G (2015). Direct conversion of normal and Alzheimer’s disease human fibroblasts into neuronal cells by small molecules. Cell Stem Cell.

[CR28] Izquierdo JM, Majos N, Bonnal S, Martinez C, Castelo R, Guigo R, Bilbao D, Valcarcel J (2005). Regulation of Fas alternative splicing by antagonistic effects of TIA-1 and PTB on exon definition. Mol Cell.

[CR29] Kansanen E, Kuosmanen SM, Leinonen H, Levonen AL (2013). The Keap1-Nrf2 pathway: mechanisms of activation and dysregulation in cancer. Redox Biol.

[CR30] Knoch KP, Bergert H, Borgonovo B, Saeger HD, Altkruger A, Verkade P, Solimena M (2004). Polypyrimidine tract-binding protein promotes insulin secretory granule biogenesis. Nat Cell Biol.

[CR31] Lander ES, Linton LM, Birren B, Nusbaum C, Zody MC, Baldwin J, Devon K, Dewar K, Doyle M, FitzHugh W, Funke R, Gage D, Harris K, Heaford A, Howland J, Kann L, Lehoczky J, LeVine R, McEwan P, McKernan K, Meldrim J, Mesirov JP, Miranda C, Morris W, Naylor J, Raymond C, Rosetti M, Santos R, Sheridan A, Sougnez C, Stange-Thomann Y, Stojanovic N, Subramanian A, Wyman D, Rogers J, Sulston J, Ainscough R, Beck S, Bentley D, Burton J, Clee C, Carter N, Coulson A, Deadman R, Deloukas P, Dunham A, Dunham I, Durbin R, French L, Grafham D, Gregory S, Hubbard T, Humphray S, Hunt A, Jones M, Lloyd C, McMurray A, Matthews L, Mercer S, Milne S, Mullikin JC, Mungall A, Plumb R, Ross M, Shownkeen R, Sims S, Waterston RH, Wilson RK, Hillier LW, McPherson JD, Marra MA, Mardis ER, Fulton LA, Chinwalla AT, Pepin KH, Gish WR, Chissoe SL, Wendl MC, Delehaunty KD, Miner TL, Delehaunty A, Kramer JB, Cook LL, Fulton RS, Johnson DL, Minx PJ, Clifton SW, Hawkins T, Branscomb E, Predki P, Richardson P, Wenning S, Slezak T, Doggett N, Cheng JF, Olsen A, Lucas S, Elkin C, Uberbacher E, Frazier M, Gibbs RA, Muzny DM, Scherer SE, Bouck JB, Sodergren EJ, Worley KC, Rives CM, Gorrell JH, Metzker ML, Naylor SL, Kucherlapati RS, Nelson DL, Weinstock GM, Sakaki Y, Fujiyama A, Hattori M, Yada T, Toyoda A, Itoh T, Kawagoe C, Watanabe H, Totoki Y, Taylor T, Weissenbach J, Heilig R, Saurin W, Artiguenave F, Brottier P, Bruls T, Pelletier E, Robert C, Wincker P, Smith DR, Doucette-Stamm L, Rubenfield M, Weinstock K, Lee HM, Dubois J, Rosenthal A, Platzer M, Nyakatura G, Taudien S, Rump A, Yang H, Yu J, Wang J, Huang G, Gu J, Hood L, Rowen L, Madan A, Qin S, Davis RW, Federspiel NA, Abola AP, Proctor MJ, Myers RM, Schmutz J, Dickson M, Grimwood J, Cox DR, Olson MV, Kaul R, Raymond C, Shimizu N, Kawasaki K, Minoshima S, Evans GA, Athanasiou M, Schultz R, Roe BA, Chen F, Pan H, Ramser J, Lehrach H, Reinhardt R, McCombie WR, de la Bastide M, Dedhia N, Blöcker H, Hornischer K, Nordsiek G, Agarwala R, Aravind L, Bailey JA, Bateman A, Batzoglou S, Birney E, Bork P, Brown DG, Burge CB, Cerutti L, Chen HC, Church D, Clamp M, Copley RR, Doerks T, Eddy SR, Eichler EE, Furey TS, Galagan J, Gilbert JG, Harmon C, Hayashizaki Y, Haussler D, Hermjakob H, Hokamp K, Jang W, Johnson LS, Jones TA, Kasif S, Kaspryzk A, Kennedy S, Kent WJ, Kitts P, Koonin EV, Korf I, Kulp D, Lancet D, Lowe TM, McLysaght A, Mikkelsen T, Moran JV, Mulder N, Pollara VJ, Ponting CP, Schuler G, Schultz J, Slater G, Smit AF, Stupka E, Szustakowki J, Thierry-Mieg D, Thierry-Mieg J, Wagner L, Wallis J, Wheeler R, Williams A, Wolf YI, Wolfe KH, Yang SP, Yeh RF, Collins F, Guyer MS, Peterson J, Felsenfeld A, Wetterstrand KA, Patrinos A, Morgan MJ, de Jong P, Catanese JJ, Osoegawa K, Shizuya H, Choi S, Chen YJ, Szustakowki J (2001). Initial sequencing and analysis of the human genome. Nature.

[CR32] Li Q, Zheng S, Han A, Lin CH, Stoilov P, Fu XD, Black DL (2014). The splicing regulator PTBP2 controls a program of embryonic splicing required for neuronal maturation. Elife.

[CR33] Li X, Zuo X, Jing J, Ma Y, Wang J, Liu D, Zhu J, Du X, Xiong L, Du Y, Xu J, Xiao X, Wang J, Chai Z, Zhao Y, Deng H (2015). Small-molecule-driven direct reprogramming of mouse fibroblasts into functional neurons. Cell Stem Cell.

[CR34] Licatalosi DD, Yano M, Fak JJ, Mele A, Grabinski SE, Zhang C, Darnell RB (2012). Ptbp2 represses adult-specific splicing to regulate the generation of neuronal precursors in the embryonic brain. Genes Dev.

[CR35] Lillevali K, Kulla A, Ord T (2001). Comparative expression analysis of the genes encoding polypyrimidine tract binding protein (PTB) and its neural homologue (brPTB) in prenatal and postnatal mouse brain. Mech Dev.

[CR36] Lin CH, Patton JG (1995). Regulation of alternative 3′ splice site selection by constitutive splicing factors. RNA.

[CR37] Linares AJ, Lin CH, Damianov A, Adams KL, Novitch BG, Black DL (2015). The splicing regulator PTBP1 controls the activity of the transcription factor Pbx1 during neuronal differentiation. eLife.

[CR38] Liu Y, Miao Q, Yuan J, Han S, Zhang P, Li S, Rao Z, Zhao W, Ye Q, Geng J, Zhang X, Cheng L (2015). Ascl1 converts dorsal midbrain astrocytes into functional neurons *in vivo*. J Neurosci.

[CR39] Liu Z, Wang L, Welch JD, Ma H, Zhou Y, Vaseghi HR, Yu S, Wall JB, Alimohamadi S, Zheng M, Yin C, Shen W, Prins JF, Liu J, Qian L (2017). Single-cell transcriptomics reconstructs fate conversion from fibroblast to cardiomyocyte. Nature.

[CR40] Llorian M, Schwartz S, Clark TA, Hollander D, Tan LY, Spellman R, Gordon A, Schweitzer AC, de la Grange P, Ast G, Smith CW (2010). Position-dependent alternative splicing activity revealed by global profiling of alternative splicing events regulated by PTB. Nat Struct Mol Biol.

[CR41] Makeyev EV, Zhang J, Carrasco MA, Maniatis T (2007). The MicroRNA miR-124 promotes neuronal differentiation by triggering brain-specific alternative pre-mRNA splicing. Mol Cell.

[CR42] Mathupala SP (2009). Delivery of small-interfering RNA (siRNA) to the brain. Expert Opin Ther Pat.

[CR43] Mitchell SA, Spriggs KA, Bushell M, Evans JR, Stoneley M, Le Quesne JP, Spriggs RV, Willis AE (2005). Identification of a motif that mediates polypyrimidine tract-binding protein-dependent internal ribosome entry. Genes Dev.

[CR44] Moreira A, Takagaki Y, Brackenridge S, Wollerton M, Manley JL, Proudfoot NJ (1998). The upstream sequence element of the C2 complement poly(A) signal activates mRNA 3′ end formation by two distinct mechanisms. Genes Dev.

[CR45] Niu W, Zang T, Zou Y, Fang S, Smith DK, Bachoo R, Zhang CL (2013). *In vivo* reprogramming of astrocytes to neuroblasts in the adult brain. Nat Cell Biol.

[CR46] Oberstrass FC, Auweter SD, Erat M, Hargous Y, Henning A, Wenter P, Reymond L, Amir-Ahmady B, Pitsch S, Black DL, Allain FH (2005). Structure of PTB bound to RNA: specific binding and implications for splicing regulation. Science.

[CR47] Patton JG, Mayer SA, Tempst P, Nadal-Ginard B (1991). Characterization and molecular cloning of polypyrimidine tract-binding protein: a component of a complex necessary for pre-mRNA splicing. Genes Dev.

[CR48] Pautz A, Linker K, Hubrich T, Korhonen R, Altenhofer S, Kleinert H (2006). The polypyrimidine tract-binding protein (PTB) is involved in the post-transcriptional regulation of human inducible nitric oxide synthase expression. J Biol Chem.

[CR49] Polydorides AD, Okano HJ, Yang YY, Stefani G, Darnell RB (2000). A brain-enriched polypyrimidine tract-binding protein antagonizes the ability of Nova to regulate neuron-specific alternative splicing. Proc Natl Acad Sci USA.

[CR50] Schoch KM, Miller TM (2017). Antisense oligonucleotides: translation from mouse models to human neurodegenerative diseases. Neuron.

[CR51] Sharma S, Falick AM, Black DL (2005). Polypyrimidine tract binding protein blocks the 5′ splice site-dependent assembly of U2AF and the prespliceosomal E complex. Mol Cell.

[CR52] Shibasaki T, Tokunaga A, Sakamoto R, Sagara H, Noguchi S, Sasaoka T, Yoshida N (2013). PTB deficiency causes the loss of adherens junctions in the dorsal telencephalon and leads to lethal hydrocephalus. Cereb Cortex.

[CR53] Singh R, Valcarcel J, Green MR (1995). Distinct binding specificities and functions of higher eukaryotic polypyrimidine tract-binding proteins. Science.

[CR54] Singh SK, Kagalwala MN, Parker-Thornburg J, Adams H, Majumder S (2008). REST maintains self-renewal and pluripotency of embryonic stem cells. Nature.

[CR55] Spellman R, Smith CW (2006). Novel modes of splicing repression by PTB. Trends Biochem Sci.

[CR56] Spellman R, Llorian M, Smith CW (2007). Crossregulation and functional redundancy between the splicing regulator PTB and its paralogs nPTB and ROD1. Mol Cell.

[CR57] Su Z, Niu W, Liu ML, Zou Y, Zhang CL (2014). *In vivo* conversion of astrocytes to neurons in the injured adult spinal cord. Nat Commun.

[CR58] Torper O, Pfisterer U, Wolf DA, Pereira M, Lau S, Jakobsson J, Bjorklund A, Grealish S, Parmar M (2013). Generation of induced neurons via direct conversion *in vivo*. Proc Natl Acad Sci USA.

[CR59] Vierbuchen T, Wernig M (2012). Molecular roadblocks for cellular reprogramming. Mol Cell.

[CR60] Vuong CK, Black DL, Zheng S (2016). The neurogenetics of alternative splicing. Nat Rev Neurosci.

[CR61] Vuong JK, Lin CH, Zhang M, Chen L, Black DL, Zheng S (2016). PTBP1 and PTBP2 serve both specific and redundant functions in neuronal Pre-mRNA splicing. Cell Rep.

[CR62] Wang J, Pederson T (1990). A 62,000 molecular weight spliceosome protein crosslinks to the intron polypyrimidine tract. Nucleic Acids Res.

[CR63] Whitman SA, Long M, Wondrak GT, Zheng H, Zhang DD (2013). Nrf2 modulates contractile and metabolic properties of skeletal muscle in streptozotocin-induced diabetic atrophy. Exp Cell Res.

[CR64] Wollerton MC, Gooding C, Wagner EJ, Garcia-Blanco MA, Smith CW (2004). Autoregulation of polypyrimidine tract binding protein by alternative splicing leading to nonsense-mediated decay. Mol Cell.

[CR65] Xue Y, Zhou Y, Wu T, Zhu T, Ji X, Kwon YS, Zhang C, Yeo G, Black DL, Sun H, Fu XD, Zhang Y (2009). Genome-wide analysis of PTB-RNA interactions reveals a strategy used by the general splicing repressor to modulate exon inclusion or skipping. Mol Cell.

[CR66] Xue Y, Ouyang K, Huang J, Zhou Y, Ouyang H, Li H, Wang G, Wu Q, Wei C, Bi Y, Jiang L, Cai Z, Sun H, Zhang K, Zhang Y, Chen J, Fu XD (2013). Direct conversion of fibroblasts to neurons by reprogramming PTB-regulated microRNA circuits. Cell.

[CR67] Xue Y, Qian H, Hu J, Zhou B, Zhou Y, Hu X, Karakhanyan A, Pang Z, Fu XD (2016). Sequential regulatory loops as key gatekeepers for neuronal reprogramming in human cells. Nat Neurosci.

[CR68] Yamamoto H, Tsukahara K, Kanaoka Y, Jinno S, Okayama H (1999). Isolation of a mammalian homologue of a fission yeast differentiation regulator. Mol Cell Biol.

[CR69] Yeo M, Lee SK, Lee B, Ruiz EC, Pfaff SL, Gill GN (2005). Small CTD phosphatases function in silencing neuronal gene expression. Science.

[CR70] Yoo AS, Sun AX, Li L, Shcheglovitov A, Portmann T, Li Y, Lee-Messer C, Dolmetsch RE, Tsien RW, Crabtree GR (2011). MicroRNA-mediated conversion of human fibroblasts to neurons. Nature.

[CR71] Zheng S, Gray EE, Chawla G, Porse BT, O’Dell TJ, Black DL (2012). PSD-95 is post-transcriptionally repressed during early neural development by PTBP1 and PTBP2. Nat Neurosci.

